# The impact of COVID-19 on mental health outcomes among hospital fever clinic attendants across Nepal: A cross-sectional study

**DOI:** 10.1371/journal.pone.0248684

**Published:** 2021-03-22

**Authors:** Hridaya Raj Devkota, Tula Ram Sijali, Ramji Bogati, Meraj Ahmad, Karuna Laxmi Shakya, Pratik Adhikary

**Affiliations:** 1 Community Support Association of Nepal (COSAN), Kathmandu, Nepal; 2 Manipal College of Medical Sciences, Pokhara, Nepal; 3 Central Institute of Science and Technology (CIST) College (Affiliated to Pokhara University), Pokhara, Nepal; 4 UC Berkeley/Institute for Social and Environmental Research, ISER-N, Bharatpur, Nepal; Iwate Medical University, JAPAN

## Abstract

**Background:**

The COVID-19 pandemic has been creating a panic and distressing situations among the entire population globally including Nepal. No study has been conducted assessing the psychological impact of this pandemic on the general public in Nepal. The objective of this study is to assess the mental health status during COVID-19 outbreak and explore the potential influencing factors among the population attending the hospital fever clinics with COVID–19 symptoms.

**Methods:**

A cross-sectional survey was conducted between May—June, 2020 with a sample of 645 participants aged 18 and above in 26 hospitals across Nepal. Telephone interviews were conducted using a semi-structured questionnaire along with a validated psychometric tool, the Depression, Anxiety and Stress (DASS-21) scale. The metrics and scores of symptoms and their severity were created and analyzed. Multivariate logistic regression was used to determine the association of potential covariates with outcome variables.

**Results:**

The prevalence of anxiety, depression and stress were 14%, 7% and 5% respectively. In reference to Karnali, participants from Bagmati province reported higher level of anxiety (OR 3.44, 95% CI 1.31–9.06), while stress (OR 4.27, 95% CI 1.09–18.32) and depressive symptoms (OR 3.11, 95% CI 1.05–9.23) observed higher among the participants in Province 1. Women were more at risk of anxiety (OR 3.41, 95% CI 1.83–6.36) than men. Similarly, people currently living in rented houses reported more stress (OR 2.97, 95% CI 1.05–8.43) and those living far from family reported higher rates of depressive symptoms (OR 3.44, 95% CI 1.03–11.46).

**Conclusion:**

The study identified increased prevalence of stress, anxiety and depressive symptoms during the initial stage of COVID-19 pandemic in Nepal. Considering the findings, there is urgent need to develop and implement appropriate community-based mental health programs targeting individuals who have had COVID-19 symptoms and who are prone to develop adverse mental health outcomes.

## Introduction

The World Health Organization (WHO) reports that as of June 26, 2020 (08:07 GMT), worldwide Covid-19 has killed 492,085 with a total of 9,724,146 individuals confirmed infected [1], and the death toll is still rising. The scale and severity of the COVID-19 pandemic has threatened public health globally. The world has been reeling, even with high income countries in havoc as a result of the global spread of this potentially fatal disease. The WHO declared COVID-19, a Public Health Emergency on 30th January 2020 a month after the outbreak of the virus in Wuhan, China alerting the global community with particular concern to the high risk countries having poor health systems [1]. Nepal in an example of a country that lacks adequate resources to tackle the COVID-19 outbreak. The government has not been able to assure the public that they are capable of handling the situation, and this has been created panic and distress throughout the entire population [2].

Nepal detected the first case of corona virus infection on 23rd January 2020 and the second case two months later, on 23rd March 2020 that surged to 11,700 affecting all 77 districts across the country with a total 28 reported deaths from COVID-19 by the end of June 2020 [3–5]. The government strategy included a country-wide lockdown to prevent a widespread outbreak of the disease and this came into effect on 24th March 2020. This lockdown was partially lifted on 14th June 2020 [6]. In addition to the illness itself, the entire population particularly the middle and low-income groups are already seriously affected through COVID-19 related issues such as lost jobs, restricted mobility and loss of freedom due to the nationwide lockdown as well as the on-going fear of disease susceptibility [7]. Moreover, the government’s poor risk communication mechanisms and the strong influence of incorrect and misleading social media rumors has been creating further terror [8]. All of these factors have negative mental health impacts on the public.

Previous studies that assessed the psychosocial impact of epidemics or pandemics such as SARS and COVID-19 found high levels of mental distress including panic attacks, and psychotic symptoms among healthcare workers and the general public [9–12]. Evidence also shows that in addition to the stress of high numbers of people getting sick or dying, epidemics and pandemics also cause vast economic losses which are associated with further high psychosocial risk [13, 14]. It should also be noted that the most vulnerable groups–people who are poor, women, children, the elderly, persons with disability and the homeless, are reported to suffer during these public health emergencies and have the greatest difficulty rebuilding their means of subsistence and social support networks after such catastrophes [10, 15]. The effects on mental health are usually more marked among populations living under precarious circumstances, who have limited resources, and limited access to social support and healthcare services. The recent studies conducted in different settings during the COVID-19 pandemics reported comparable findings, with the highest levels of distress among women, rural inhabitants, elderly populations, groups with lower levels of education, migrant workers and those having unstable incomes [16, 17]. Moreover, perceived disease susceptibility and perceived disease severity [18], social isolation, and spending longer time watching COVID-19 related news and social media are also found associated risk factors with increased level of mental distress [19]. WHO reports that the burden of distress, depression and other mental health conditions such as suicide is on the rise globally. It further reports that long-lasting moderate or severe depression related to COVID-19 pandemic may become a serious public health concern [20].

The burden of COVID-19 related mental disorders continues to grow with significant impacts on health and major social, human rights and economic consequences globally. Furthermore, mental health disorders, fear-related behaviors, stigmatization, and negative effect on access and quality of care during and in the aftermath of the epidemics are commonly reported [21, 22]. In summary, COVID-19 has the potential to create devastating social, economic and mental health crises which may have long-term impact, particularly in a country like Nepal.

In light of this, it is urgent to understand the current level of anxiety and stress due to COVID-19 in Nepal and to recommend evidence based mental health intervention policies to cope with this efficiently. It is further urgent that evidence-driven strategies be developed to reduce adverse psychological impacts and psychiatric symptoms during and after COVID-19. This study is intended to contribute to this effort by identifying potential factors that affect the mental health status of fever clinics patients with the symptoms of COVID–19. We hypothesized that the prevalence and levels of depression and anxiety as indicated on the respective Depression, Anxiety, and Stress Scale (DASS) subscales may be elevated among the study population.

## Methodology

### Study design and population

This study is a cross-sectional survey, conducted among the fever clinic attendants with symptoms of COVID-19 across various health care facilities in Nepal. The survey was conducted between May 17 to June 9, 2020 during the nationwide lock-down that started on 24^th^ March 2020.

### Participants’ recruitment procedure

**Fig 1:**
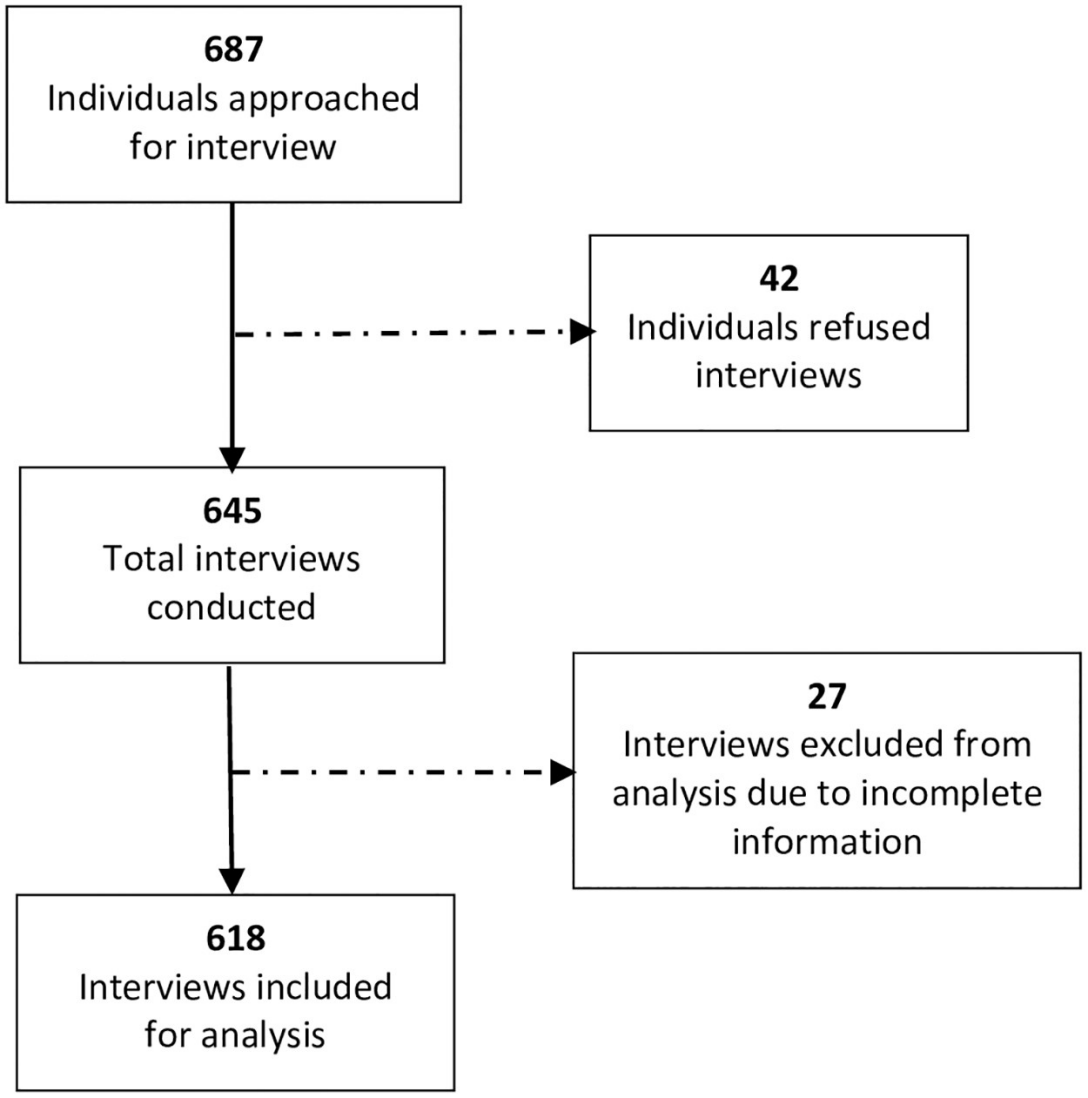
Flowchart of the sampling design and subject enrolment in the study.

The study covered all seven provinces across Nepal. A multi-stage sampling method was used for the selection and recruitment of participants. Twenty-six health facilities (hospitals and primary healthcare centres) from 23 districts were purposively selected ranging between 3–6 health facilities from each province covering both ecological zones, Hills and Terai. Hospitals or primary health care centres who had fever clinics and showed their interest to participate in the study were the key criteria for selecting health facilities. A sampling frame was developed collecting the names of those who attended hospital fever clinics between April 25 and May 16, 2020. Out of the total 1285 fever clinic attendants during the period, 687 met the study’s eligibility criteria and included in the final list for interviews. Individuals aged 18 and above, who visited hospital suspecting or having COVID-19 symptoms were inclusion criteria set for the study. The refusal rate for interviews was 6%. Fig 1 presents the number of participants approached for interviews and inclusion exclusion criteria used the study.

### Survey instrument and data collection procedure

A semi-structured questionnaire with socio-demographic information was used along with a 21-items depression, anxiety and stress scale (DASS-21)–a set of 3 self-reporting scales developed by Lovibond & Lovibond, 1995 [23] to measure the emotional states of depression, anxiety and stress were administered by the trained interviewers. The tool’s overall reliability coefficient (Cronbach’s Alpha) estimated 0.90. While estimating separately, it was 0.71, 0.79 and 0.77 for Depression, Anxiety and Stress respectively. The tool has already been tested in Nepal, its psychometric properties validated and it was found to be simple, easy to administer, and simple to score. It has been used extensively in previous studies globally as well as in Nepal [24–26].

Both the standardized questionnaire and DASS-21, were set up on tablet computers and mobile phones with KoBo Collect software, and administered in Nepali through telephone interviews by trained data collectors. The questionnaire was first developed in English, translated into Nepali by three bilingual Nepalese and field-tested for acceptability and comprehension among the population in which it was to be used. On average, administration of the questionnaire took 24 minutes.

### Ethical approval

The researchers obtained ethical approval from the Nepal Health Research Council (NHRC)—ERB Protocol Registration No. 317/2020P. Before interviews, verbal informed consent was taken from all participants.

### Measures

DASS-21 score was the outcome variable that ranged from 0–42. The participant’s reaction to each statement was measured in a response category ranging from 0–3 to indicate “did not apply to me at all” to “applied to me very much most of the time”. Following the DASS– 21 scoring instructions, the sum of the rated scores in each statement was multiplied by 2 to calculate the final score [23]. The cut-off (threshold) scores for detecting stress, anxiety and depression were 15, 8 and 10 respectively. The severity levels categorised as normal, mild, moderate and severe, and their score ranged as stated in the table below (Table 1).

**Table 1:** Severity level and score ranges.

Severity level	Stress	Anxiety	Depression
Normal	0–14	0–7	0–9
Mild	15–18	8–9	10–13
Moderate	19–25	10–14	14–20
Severe	26 and above	15 and above	21 and above

Socio-demographic data were self-reported by the participants. The knowledge and perceived risk indicators were created by 27 and 6 questionnaire items respectively to derive scores. The questionnaire included the questions related to COVID-19 symptoms, the risk groups, mode of transmission and prevention for knowledge assessment, while the individual’s belief about remaining safe from COVID-19, easy availability of healthcare services, and belief about government’s ability to control and overcome the pandemic was asked for risk perception. All the questionnaire items were equally weighted, dichotomized, and a composite measure was created using the sum with the maximum scores of 27 and 6 respectively.

Table 2 provides the variables and their definition used in the study.

**Table 2:** Variables and their definitions used in the study.

Variables	Definition
***Outcome Variables***	
Stress, Anxiety and Depression	Total algebraic sum of the rated scores by the respondents (between 0–42)
***Covariates***	
Province	Administrative division reported by the respondents as their permanent address.
Ecological region	The ecological (Hills or Terai) region of the respondent’s habitation.
Place of residence	Respondent’s place of residence–rural or urban at the time of survey.
Age	Completed age in years of respondent at the time of interview.
Gender	Self-reported sex identity of respondent—male or female)
Caste and ethnicity	Self-reported caste and ethnic group—Brahmin/Chhetri, Jana Jaati, Dalit, Madhesi, Muslims and others.
Religion	Self-reported religious belief of the respondent—Hinduism, Buddhism, Others.
Education	Number of years of education completed—no formal education, primary education, secondary education, higher education.
Primary occupation	Main occupation of the respondent at the time of interview—labor & others, service, foreign employment, farming, business and self-employment.
Marital status	Self-reported marital status of the respondent at the time of survey—married, ever married, single (widowed or divorced)
Family type	Husband, wife including the children considered a nuclear family, more than those living together is defined as joint or extended family.
Have own house	Respondent having his/her own house in any district or province—Yes or No
Current living	Respondent living (at his/her own house or rented house) at the time of survey
Living with family?	Respondent living with the family at the time of survey—Yes or No
Travel history	Recent travel outside the country—Yes or No
Health condition	Self-rated health status at the time of survey—very good, good, fair and poor.
Knowledge	Knowledge of symptoms, risk, transmission, and prevention spontaneously cited relating to COVID 19 presented as an additive score/index. It adapted 9 items related to symptom, 2 items risk, and 8 items each for transmission and prevention.
Perceived risk	An index developed using 6 questionnaire items at individual beliefs on safety, availability of health services, and government ability controlling the pandemic—nominal, medium, high.

Note: The reliability coefficient (Cronbach’s Alpha) of 27-item knowledge questionnaire was estimated at 0.80.

### Statistical analysis

The data collected in KoBo Collect software were downloaded into Excel Windows 10, cleaned and then transferred into SPSS (version 23.0 for Windows) for analysis. Descriptive statistics and associations between the outcome and potential covariates were examined using bivariate odds ratios (ORs) and 95% confidence intervals (CI). Due to the binary nature of the dependent variable, unconditional logistic regression was used. Any variable associated with the outcome variable with a p-value <0.2 in the bivariate analysis were further investigated for confounding by multivariate logistic regression model examining associations of potential covariates with outcome variables (stress, anxiety and depression) [27].

## Results

### Characteristics of study participants

Out of 687 participants interviewed, a total of 618 interviews with complete information were included in the analysis. Of these, the highest proportion of participants (17.8%) were from Karnali (Province 6) and the lowest from Province 5. The majority of study participants (79%) lived in urban area, while 63.6% in the hills. The average age of the participants was 35 years, with ages ranging from 18–85 (SD = 14.25). Over one-third (37%) were women, over half (55%) reported having secondary level education and 16% with higher education. Nearly 4 in 10 reported their occupation as laborers and 16.8% reported having foreign employment. The vast majority of participants (86.1%) reported their religious belief as Hindu, with 42.71% and 17.8% reporting their caste group as Brahmin/Chhetri and Dalits respectively. More than 93% of respondents reported having their own house, however, only 83% were living in their house at the time of survey. About 77% of participants were married and 93% were living together with their family at the time of survey (Table 4).

### Prevalence and severity measurement of stress, anxiety and depression

A significant proportion of participants (14%) had symptoms of anxiety, while 7% and 5% reported depression and stress respectively. Over 5% had mild, 6% had moderate and just under 3% had severe levels of anxiety. Similarly, 3.4% had mild depression and 2.8% and 0.6% had the moderate and severe depressive symptoms. Under 3% had a mild level of stress and only 1.1% had moderate and another 1.3% had a severe level of stress (Table 3).

**Table 3:** Prevalence and severity of stress, anxiety and depression.

Severity level (Score)	Stress		Anxiety		Depression	
	n = 618	%	n = 618	%	n = 618	%
Normal (0–14)	587	95.0%	533	86.2%	576	93.2%
Mild (15–18)	16	2.6%	33	5.3%	21	3.4%
Moderate (19–25)	7	1.1%	37	6.0%	17	2.8%
Severe (26 +)	8	1.3%	15	2.4%	4	0.6%

Table 4 displays the result of the bi-variate logistic regression analysis. Among the participants across seven provinces, anxiety was found more prevalent in Bagmati (OR 3.79, 95% CI 1.60–8.98) and Sudurpaschim (OR 2.71, 95% CI 1.08–6.80) compared to Karnali. However, both stress and depression were reported as higher in Province 1 with OR 4.84, 95% CI 1.31–17.93 and OR 2.87 95% CI 1.12–7.37 respectively. Health condition, age, gender and marital status all showed the positive association with anxiety. The odds for those with poor health conditions (OR 3.72, 95% CI 1.08–12.83), the age group over 55 compared to the younger age (OR 2.86, 95% CI 1.35–6.07), women compared to men (OR 3.20, 95% CI 1.99–5.13) and divorced or widowed individuals compared to those with a partner (OR 5.00, 95% CI 1.80–13.88) were higher. Education also showed the positive association with anxiety (P = 0.01). Participants with secondary level education had lower odds (OR 0.47 95% CI 0.28–0.79) compared to those who had primary or lower level education. Individuals living in a rented house reported being more likely to develop both stress (OR 2.50, 95% CI 1.14–5.47) and anxiety (OR 1.78, 95% CI 1.03–3.07) than those living in their own house. Similarly, individuals living separately from their families reported having more stress compared to those living with their families (OR 2.70, 95% CI 0.98–7.42). Participants who recently travelled abroad were less likely to have anxiety compared to those who did not travel (OR 0.47, 95% CI 0.24–0.91).

**Table 4:** Socio-demographic characteristics of the study participants with bivariate Odds Ratios (ORs).

Covariate	Frequency (%)	Stress			Anxiety			Depression		
	N = 618	OR	95% CI	*P*-Value	OR	95% CI	*P*-Value	OR	95% CI	*P*-Value
**Province**										
Karnali (6)	110 (17.8%)	01:00			01:00			1		0.22
Gandaki (4)	107 (17.3%)	0.68	0.11–4.15	0.68	2.08	0.84–5.13	0.11	0.42	0.11–1.69	0.59
Bagmati (3)	96 (15.5%)	3.69	0.97–14.05	0.06	3.79	1.60–8.98	0.002	1.34	0.47–3.84	0.03
Province (1)	92 (14.9%)	4.84	1.31–17.93	0.02	1.56	0.59–4.12	0.37	2.87	1.12–7.37	0.12
Sudurpaschhim (7)	80 (12.9%)	0.45	0.05–4.42	0.5	2.71	1.08–6.80	0.03	0.19	0.02–1.55	0.67
Province (2)	75 (12.1%)	2.01	0.44–9.25	0.37	1.96	0.74–5.23	0.18	1.28	0.41–3.97	0.43
Province (5)	58 (9.4%)	0.63	0.06–6.15	0.69	1.47	0.49–4.46	0.5	0.53	0.11–2.62	
**Ecological area**										
Hills	393 (63.6%)	1			1			1		
Tarai	225 (36.4%)	1.11	0.53–2.33	0.79	0.79	0.48–1.29	0.34	1.2	o.64–2.28	0.57
**Place of residence**										
Urban	488 (79%)	1			1			1		
Rural	130 (21%)	0.54	0.19–1.58	0.26	1.37	0.81–2.33	0.24	0.74	0.32–1.70	0.47
**Age**										
18–24	151 (24.4%)	1			1			1		
25–34	221 (35.8%)	1.98	0.70–5.60	0.2	1.48	0.77–2.85	0.24	1.3	0.54–3.15	0.56
35–44	123 (19.9%)	1.76	0.55–5.70	0.34	1.17	0.54–2.52	0.7	1.76	0.68–4.51	0.24
45–54	48 (7.8%)	0.62	0.07–5.45	0.67	1.55	0.59–4.05	0.37	1.19	0.30–4.68	0.8
55 +	75 (12.1%)	1.65	0.43–6.32	0.47	2.86	1.35–6.07	0.01	1.28	0.40–4.05	0.68
**Gender**										
Male	390 (63.1%)	1			1			1		
Female	288 (36.9%)	1.44	0.69–2.97	0.33	3.2	1.99–5.13	0	1.78	0.95–3.34	0.07
**Caste and Ethnicity**										
Brahmin/Chhetri	264 (42.7%)	1			1			1		
Jana Jaati	160 (25.9%)	0.99	0.42–2.32	0.98	0.69	0.38–1.25	0.22	1.71	0.79–3.69	0.17
Dalit	110 (17.8%)	0.15	0.02–1.17	0.07	0.86	0.45–1.63	0.64	1.03	0.39–2.75	0.95
Madhesi/Muslims	84 (13.6%)	1.28	0.48–3.40	0.63	0.82	0.40–1.68	0.59	1.88	0.76–4.65	0.17
**Religion**										
Hinduism	532 (86.1%)	1			1			1		
Buddhism	48 (7.8%)	0.81	0.19–3.53	0.78	0.55	0.19–1.59	0.27	1.82	0.67–4.90	0.24
Others	38 (6.1%)	1.04	0.24–4.55	0.96	1.14	0.46–2.83	0.77	2.37	0.87–6.48	0.09
**Education**										
Primary or less (<Grade 5)	182 (29.5%)	1			1			1		
Secondary (6–12)	340 (55.0%)	1.01	0.42–2.41	0.99	0.47	0.28–0.79	0.01	0.65	0.33–1.29	0.21
Higher education	96 (15.5%)	1.98	0.72–5.45	0.19	1.01	0.53–1.89	0.99	0.69	0.26–1.83	0.46
**Primary Occupation**										
Labor	240 (38.8%)	1			1			1		
Service	106 (17.2%)	1.14	0.38–3.42	0.82	0.77	0.39–1.51	0.44	0.41	0.12–1.43	0.16
Foreign employment	104 (16.8%)	1.41	0.50–3.98	0.52	0.58	0.28–1.22	0.15	1.49	0.65–3.40	0.35
Farming	94 (15.2%)	1.02	0.31–3.34	0.97	1.21	0.64–2.28	0.55	1.13	0.45–2.83	0.8
Business/Self-employed	74 (12.0%)	2.03	0.71–5.79	0.19	0.67	0.30–1.50	0.33	1.24	0.47–3.28	0.67
**Marital Status**										
Married	475 (76.9%)	1			1			1		
Ever Married	127 (20.6%)	1.15	0.48–2.74	0.76	0.8	0.43–1.47	0.47	0.52	0.20–1.34	0.18
Single (Widow, Divorced)	16 (2.6%)	1.31	0.17–10.35	0.8	5	1.80–13.88	0.002	1.8	0.39–8.22	0.45
**Family Type**										
Extended	451 (73.0%)	1			1			1		
Nuclear	167 (27.0%)	1.11	0.50–2.46	0.8	0.93	0.56–1.57	0.8	0.96	0.47–1.95	0.9
**Do you have own house?**										
Yes	576 (93.2%)	1			1			1		
No	42 (6.8%)	0.94	0.22–4.10	0.94	1.79	0.83–3.90	0.14	0.67	0.16–2.87	0.59
**Currently living @**										
Own house	514 (83.2%)	1			1			1		
Rented	104 (16.8%	2.5	1.14–5.47	0.02	1.78	1.03–3.07	0.04	1.18	0.53–2.62	0.69
**Living with Family**										
Yes	574 (92.9%)	1			1			1		
No	44 (7.1%)	2.7	0.98–7.42	0.05	1.96	0.93–4.13	0.08	2.36	0.94–5.95	0.07
**Travel History**										
No	479 (77.5%)	1			1			1		
Yes	139 (22.5%)	0.82	0.33–2.04	0.67	0.47	0.24–0.91	0.03	1.08	0.52–2.26	0.83
**Knowledge (score)**										
< 7	99 (16%)	1			1			1		
8–14	368 (59.5%)	1.08	0.35–3.31	0.89	0.58	0.32–1.03	0.06	0.83	0.36–1.90	0.66
15–21	140 (22.7%)	1.63	0.49–5.46	0.43	0.51	0.25–1.04	0.07	0.6	0.21–1.71	0.34
22 +	11 (1.8%)	5.28	0.85–32.90	0.08	0.88	0.18–4.39	0.87	2.53	0.47–13.76	0.28
**Self-reported health**										
Very good	100 (16.2%)	1			1			1		
Good	333 (53.9%)	0.54	0.21–1.39	0.2	0.69	0.35–1.37	0.29	0.8	0.33–1.97	0.63
Fair	171 (27.7%)	0.74	0.27–2.05	0.56	1.79	0.90–3.56	0.1	1.09	0.42–2.84	0.86
Poor	14 (2.3%)	2.21	0.41–11.91	0.35	3.72	1.08–12.83	0.04	3.62	0.82–16.08	0.09
**Perceived Risk**										
Nominal	277 (44.8%)	1			1			1		
Medium	237 (38.3%)	0.91	0.41–2.05	0.83	1.32	0.80–2.17	0.27	0.99	0.51–1.94	0.98
High	104 (16.8%)	1.15	0.43–3.08	0.78	0.9	0.45–1.81	0.77	0.65	0.24–1.78	0.4

### Factors associated with mental health outcome

After control of confounders, only the province showed significant association with all outcome variables. The adjusted odds for both stress (OR 4.27, 95% CI 1.09–18.32), and depression (OR 3.11, 95% CI 1.05–9.23) were higher among participants in province 1 than in Karnali. However, Bagmati province had higher odds for anxiety (OR 3.44, 95% CI 1.31–9.06). Gender was associated to anxiety with higher odds among female (OR 3.41, 95% CI 1.83–6.36) than male. Also, the current living status (owned or rented house) was significantly associated with stress. The odds among those living in rented houses was higher than those living at own house (OR 2.97, 95% CI 1.05–8.43). Participants living far from their family reported more depressive symptoms than those living with their families (OR 3.44, 95% CI 1.03–11.46) (Table 5).

**Table 5:** Multivariate analysis of stress, anxiety and depression by selected variables.

Covariate	Stress		Anxiety		Depression	
	OR	95% CI	OR	95% CI	OR	95% CI
**Province**						
Karnali (6)	1		1		1	
Gandaki (4)	0.39	0.06–2.73	1.25	0.45–3.50	0.35	0.08–1.52
Bagmati (3)	2.34	0.54–10.15	*3.44	1.31–9.06	1.36	0.42–4.43
Province (1)	*4.27	1.09–18.32	1.54	0.53–4.49	*3.11	1.05–9.23
Sudurpaschhim (7)	0.3	0.03–3.11	2.67	0.96–7.41	0.15	0.02–1.35
Province (2)	1.21	0.17–8.46	1.81	0.57–5.68	1.36	0.33–5.53
Province (5)	0.35	0.03–4.05	1.28	0.39–4.23	0.53	0.10–2.76
**Gender**						
Male			1	1.83–6.36		
Female			***3.41			
**Currently living @**						
Own house	1					
Rented house	*2.97	1.05–8.43				
**Living with Family**						
Yes					1	1.03–11.46
No					*3.44	

*P<0.05,

**P<0.01,

***P<0.001

## Discussion

This study found 14% of the respondents with anxiety, 7% with depression and 5% with stress symptoms. Among them, moderate–severe level of anxiety was reported by 61%, and depression and stress by 50% and 48% respectively (Table 3). The prevalence rates observed in the present study were not dramatically high compared to the recent studies conducted in other countries e.g. in China [19], and Italy [28] at the time of pandemics, however we found the prevalence of anxiety and depression higher than the estimated national prevalence rate (3.6% for anxiety and 3.2% for depression) at Nepal reported by WHO in 2017 [29]. Our findings from this study were in line with the results of many other previous studies conducted in other countries. A study conducted during lockdown in India using the same tool (DASS-21) reported anxiety, depression and stress at 10%, 11% and 13% respectively [30]. Moreover, a systematic review of COVID–19 and mental health literature conducted recently revealed the anxiety and depression prevalence between 16%– 28% and stress at 8% [10]. Another study in Italy showed very high prevalence of depression at 32% and stress at 27% [28]. The possible explanation for this wide variations in the prevalence could be the differences in the study populations, situations or the use of inconsistent definitions and instruments in the studies.

The result of the multivariate regression analysis showed that the participants in province 1 were more prone to develop an increased level of stress and depression, while participants in Bagmati province were found to be more likely to develop anxiety disorder compared to those in Karnali. There could be various explanations for this. A possible explanation may be that more knowledge and information on the spread of the virus and its potential risks was available to people in Bagmati and Province 1 since these two provinces have more access to mass media compared to Karnali [31]. Moreover, at the time of this study, the virus in Nepal was still perceived as being imported from other countries, the scope of community transmission to the rural province like Karnali was lower and the public in those area may have had yet to realize the pandemic’s scope in their territory.

Another finding from this study was that more women than men have been found to have increased levels of anxiety during the outbreak of COVID-19. This finding is in line with previous studies conducted in China [11, 16], India [30] and Italy [28], which have consistently found increased psychological distress higher among females compared to males. The finding may also be linked to evidence in the international literature that women likely to be more vulnerable to experiencing stress and developing post-traumatic symptoms. The explanation for this could be that women tend to feel increased responsibility to not only keep themselves well but maintain the health and well-being of their families–including older relatives, children and grandchildren that may have created more stress.

The other factors, such as people living at rented house and far from the families found more at risk to develop stress and depressive disorders. These people may also be poorer and at higher risk of immediate impact–with little savings or material goods they could sell if sick and unable to work, the impact of getting sick with COVID-19 could be immediate both for themselves and for members of their families.

The previous studies have identified that the advanced age population, particularly those over 60 and comorbid with poor health were at most risk contracting the disease and also had a higher mortality to COVID– 19 [17, 32]. Corresponding to this, several studies conducted during this pandemic found the age and individual health condition highly linked to mental health outcome reflecting the increased psychological distress during the COVID-19. However, contrary to this, our study did not show any differences in increased level of stress, anxiety or depression between age groups. The possible reason for this could be the age cut-off value in this study at 55 years may have remained low to show this differences.

### Strength and limitation of the study

To the best of our knowledge, this is the first population study to evaluate the mental health status among Nepalese people during the COVID-19 pandemic. This study is therefore, an important contribution to the literature as it provides preliminary data about the impact of COVID-19 on mental health in Nepal. The strength of this study is a fairly large sample (n = 618) recruited from 26 health facilities across the country representing main populations groups from different strata that included ecological region, urban rural residents and caste ethnicity. Moreover, the study had a high response rate (94%) that is considered good enough for telephone-based survey. Furthermore, the telephone interviews were taken by the well-trained and highly experienced interviewers under close supervision that ensured the quality of data collected.

With all these strengths, the study had a number of limitations. We intended to have a representative sample covering all province and ecological clusters, however, the purposive selection of health facilities conducting fever clinics may have resulted selection bias. Likewise, the potential sampling biases may have occurred with under or over representation from the different cluster, social groups, and also with the criteria as exclusion of under 18 populations and individual with communication and long-standing medical problems. Furthermore, the study population were only those who visited the fever clinic and recorded in the hospital register. However, a complete database of the clinic attending patients was not created across hospitals. Thus participants were selected purposively rather than random sampling procedure. It should also be noted that face to face interview was not possible due to lockdown and participants were interviewed over the phone on this sensitive issue. The absence of visual cues on the phone might have compromised creating comfortable environment for interview, rapport and probing [33]. Also, the possibility of social desirability bias could not be ignored since the data were self-reported and there was no means of (clinical) verification.

## Conclusion

The result of this study shows an increased prevalence rate of mental health outcomes (stress, anxiety and depressive symptoms) among the study population. Interestingly however, the increased rates observed in the present study were not intensely high compared to the findings reported in previous studies conducted in the countries highly affected by COVID-19. This is noteworthy considering that this study was implemented in the initial phase of the pandemics in Nepal.

Most importantly, the study identified the key factors contributing to adverse mental health outcome and the population groups who are potentially more vulnerable to the pandemics. However, it is difficult to draw any conclusions regarding its long-term effect due to the cross-sectional nature of the current study. Further research is needed to track whether these groups show higher levels of psychosocial distress at later stages in pandemics. Longitudinal studies are recommended for understanding the trajectories of mental health among the population during and after the pandemic of COVID-19. Also, qualitative studies could be useful to understand how people cope with the pandemic and what psychosocial supports they have or feel they need in response to the pandemic.

One key policy implication of the present study is that the government should provide psychological support to all those already affected and who are prone to develop mental health concerns, not just the symptoms but the actual health problems need to be addressed during the pandemic. The individuals who are suffering through mental distress, and prone to develop serious symptoms in later stages must be reached with medical and counseling support. More attention needs to be given to vulnerable groups such as women, people with pre-existing illnesses and disabilities, farmers, and the migrant populations living away from their homes—far from their families and support networks.

## Supporting information

S1 Data (SAV)
